# 

**Published:** 1909-04

**Authors:** 


					﻿Epistle to the Privates in the Rear Rank.
The White Feather in Evidence.
Vol. XXIV.
APRIL, 1909.
No. 10.
TEXAS
MEDICAL
JOURNAL
“RED BACK”
PUBLISHED AT AUSTIN, TEXAS, BY
F. E. DANIEL, M. D.,	-	- Proprietor
PRINTED BY THE VON BOECKMANN-JONES CO.
Subscription, $1.00 a Year, in Advance. -	Single Copies, 10 Cents
Entered at the Postoffice at Austin, Texas, as Second-class Mail Matter
				

